# Boosting electrocardiography leads location training through virtual reality simulation: effects on skill performance and learner satisfaction

**DOI:** 10.1186/s12909-026-09854-9

**Published:** 2026-07-03

**Authors:** Fatemeh Kermanian, Parham Safiyari, Gholamreza Hassanzadeh, Rita Mojtahedzadeh, Aeen Mohammadi

**Affiliations:** 1 Department of Anatomy, School of Medicine, Alborz University of Medical Sciences, Karaj, Iran; 2 Department of Computer Engineering, Faculty of Engineering, University of Kurdistan, Sanandaj, Iran; 3 Department of Anatomy, School of Medicine, Tehran University of Medical Sciences, Tehran, Iran; 4 Department of E-Learning in Medical Education, Center of Excellence for E-learning in Medical Education, School of Medicine, Tehran University of Medical Sciences, Tehran, Iran

**Keywords:** Virtual reality, Simulation, Medical education, Anatomy

## Abstract

**Background:**

The purpose of this study was to investigate the effect of electrocardiography lead placement training on the performance and satisfaction of medical students using VR and compare it with training on a real patient and training on a mannequin.

**Methods:**

This was a true experimental study. Ninety undergraduate medical students in the internal medicine rotation course were included in the study. The simulation software for the insertion of electrocardiography lead placement training was designed. The students were then divided into three groups. Practical training for lead placement was conducted via three methods: training on a real patient, a mannequin, and training through VR simulation. The course was evaluated through the DOPS (Direct Observation of Procedural Skills) test and the satisfaction survey form.

**Results:**

The analysis of the DOPS test scores revealed a significant difference between the VR group and the other two groups (patient: 15.2 (1.89), mannequin: 15.3 (1.71) and VR: 17.5 (1.35)) (P value < 0.001). The results of the survey also indicated that the satisfaction of the students in the VR group was significantly greater than that of the other two groups of patients: (0.24) 1.74, (0.18) 1.76 and (0.18) 2.35 (P value < 0.001).

**Conclusion:**

Considering the attractiveness of VR, the use of this software along with other teaching models in anatomy education can increase the motivation and satisfaction of learners.

**Supplementary Information:**

The online version contains supplementary material available at https://doi.org/10.1186/s12909-026-09854-9.

## Background

Anatomy is the most relevant basic discipline for daily clinical activity. Two common pedagogies are cadaveric dissection and examination of prosected samples. However, the number of cases in which anatomy courses are delivered through the traditional dissection mode is declining due to limited access. Technological advancements have opened new opportunities to investigate novel teaching methods. Before computers were used in medical education, textbooks, cadavers, anatomical models, and live patients were some of the only pedagogical tools available [1]. The implementation of virtual 3D (Three-Dimensional) training methods has the potential to dissolve the growing misalignment between learning and teaching in undergraduate medical curricula. Virtual reality (VR) offers benefits for learners and educators, providing cost-effective and repeatable clinical training on demand [2–4]. VR uses software to create an immersive simulated environment. Unlike traditional user interfaces, VR places the user inside an experience, where they can engage with the environment and virtual characters in a way that feels real [5]. VR is a wide notion that has different tools. There are three main categories of VR simulators: screen-based VR, immersive VR environments and virtual worlds. Screen-based VR consists of an interface connected to mechanical devices and can be displayed on any screen but most commonly uses a desktop. This type of VR has commonly been used to develop technical psychomotor skills, such as for endoscopic surgery, because it can be used repeatedly and requires very little time to set up [6]. Multiple studies have used VR-assisted surgical techniques to enhance surgical planning and navigation [7–10]. While many studies exist on the use of virtual 3D environments for teaching anatomy [11–13], their use in surface anatomy teaching is unexplored. The virtual assessment scenarios currently reviewed in the literature focus primarily on clinical skills rather than anatomy [14]. The use of VR in anatomy instruction provides two primary benefits. First, observers can freely adjust their observation viewpoint. Second, virtual anatomical structures can be disassembled and flipped for examination [15]. There is a significant tendency toward educational approaches that facilitate the application of knowledge in practice [14, 15].

Clinical placements have significantly improved students’ practical skills, but an absence of extensive practical training can often prevent their ability to master these procedures. The main goal of virtual reality is to create a sense of presence in a virtual space. The created space must be believable and sufficiently interactive to require the user to perform specific actions.

Recording the electrical activity of the heart is one of the primary procedures in emergency centers and is performed by medical staff every day. The correct placement of electrocardiography leads requires superficial anatomical knowledge. Numerous studies have shown that, in most cases, this placement is not performed correctly. The most common reasons for incorrect placement are patient obesity and a lack of familiarity with superficial signs of the chest wall. The most common errors are in the placement of leads V1 and V2 [16, 17].

Given the large crowd of students in the clinic and the inability to trial-and-error patients in clinics and teaching hospitals, we decided to design software to provide learners with the opportunity to repeatedly place leads, receive feedback, and try again in a safe environment that is completely similar to the clinic. The purpose of our study was to develop a VR-based system for electrocardiography lead placement to be used in a virtual environment. The aim was to augment the students’ spatial perception with a 3D model based on the surface anatomy of the anterior wall of the thorax. This study, therefore, aims to investigate how computer simulation in clinically oriented anatomy teaching can affect student performance and satisfaction.

## Methods

### Hardware and software

**Fig. 1:**
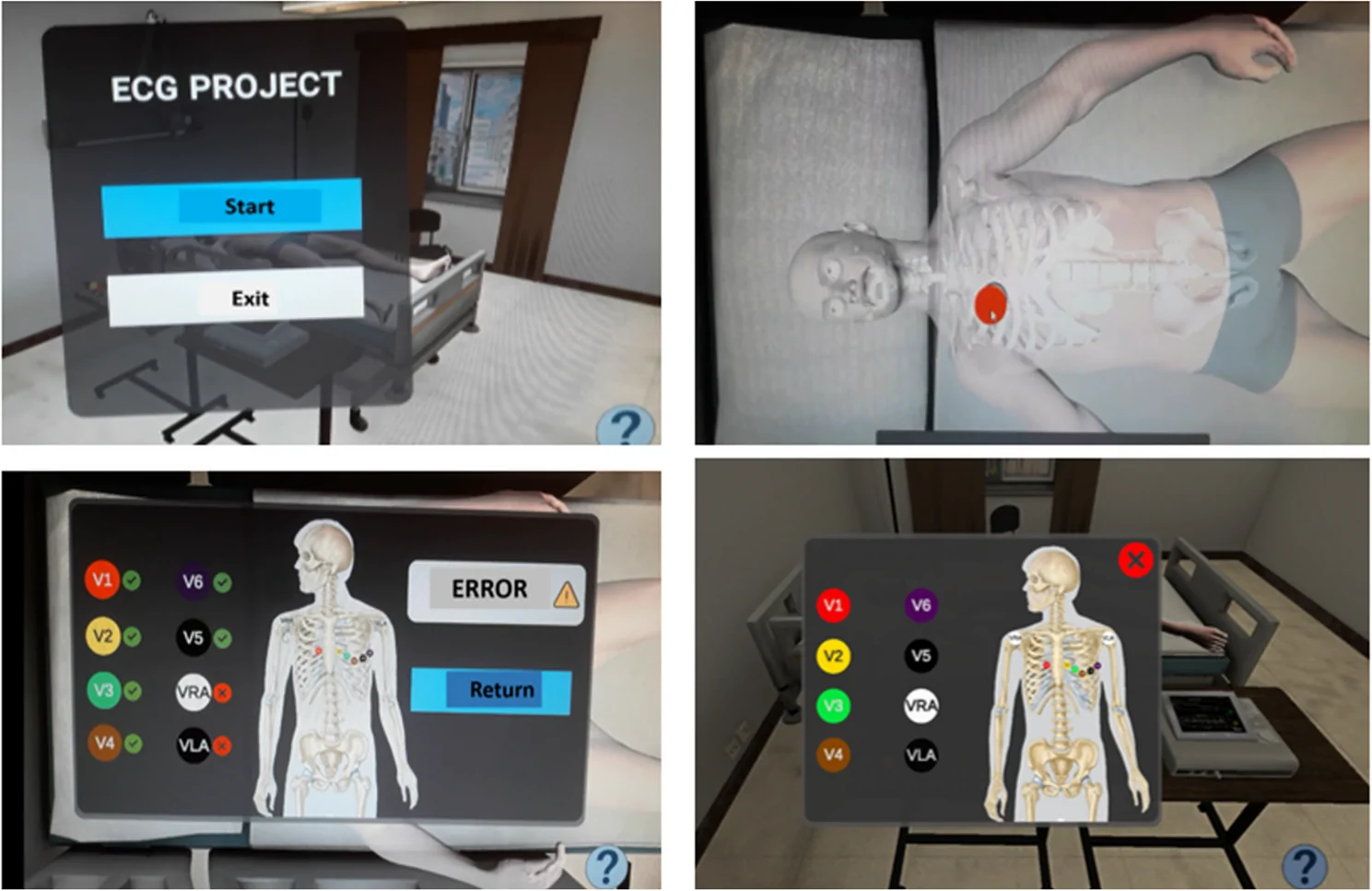
The user interacting with the VR system

A unique screen-based VR (by using a desktop) with an emphasis on the bony landmarks of the anterior thoracic wall was created. A professional software producer prepared the software and added effects (e.g., thoracic cage movements). This virtual environment was verified for anatomic correctness by an experienced professor of anatomy. The 3D simulation system provides learners with a visualized bony thoracic wall. The interactive nature supports operations such as rotate, pick up, place, and move. The VR system used for the purpose of this study uses a static monitor as a display device. This software is similar to the real simulated environment in that the virtual patient lies on his or her back on the hospital bed, and the anatomical elements of the anterior chest wall, including the ribs and sternum, are clearly defined. Next, an electrocardiogram recording device with six precordial leads and two upper limb leads can be seen. Using the mouse, the surveyor lifts each of the leads and brings it near the patient, and by observing the super-imposed elements of the thorax, the surveyor places the lead in the right place. After placing eight leads and pressing the end button, the program shows the results on the computer screen. If there is a mistake in placement, the wrong lead is identified, and the surveyor places it again. The user interacting with the VR system is shown in Fig. 1.

### Implementation of the training program

The study population consisted of all 90 fourth-year medical students who were enrolled in the cardiovascular disease course from 2023 to 2024 at Alborz University of Medical Sciences. The students were randomized into three groups via randomization software. The first group received patient-based training in the clinic. The second group received skill-lab training via a mannequin, and the last group received virtual simulation-based training via a computer. The content was the same for all groups, and the same teacher prepared it. The students had no restrictions for training in all groups and they don’t receive direct feedback from the educator. The class duration (two hours) and teacher were the same for all three groups. In VR group, their access to VR program was limited to class time.

### Assessment

Learners were evaluated through the DOPS test (Direct Observation of Procedural Skills). This test is a method specifically designed to evaluate procedural skills. In this method, the learner is observed while performing the procedure, their performance is evaluated on a checklist, and feedback is given to the learner. We used a standard DOPS test and it was applied the same to all groups in a clinical scenario on a live patient. With DOPS test, the positioning of the leads was tested. The process of observing the student took approximately 15 min, and providing feedback to them took approximately 5 min. The test was held one week after the class meeting. The evaluation checklist included five areas of activity, each of which was scaled with degrees above the expected limit, within the expected limit, borderline limit, below the expected limit, and no opinion. These areas included technical ability, knowledge of the superficial anatomy, preparation before, during and after the procedure. The teacher recorded the quality of the clinical procedure in each area. If the students do not receive the desired grade in an area, after hearing the teacher’s feedback, they should repeat the process. For the option “higher than expected”, a score of 20 was given; for “within the expected range”, a score of 17 was given; for “borderline”, a score of 14 was given; for “lower than expected”, a score of 10 was given; and for “no opinion”, a score of 7 was included. The final grade of the student was the average of the grades obtained in five fields.

### Satisfaction

At the end of the tutorial and before the DOPS test is given, the satisfaction of the students was checked through an anonymous questionnaire containing 16 explicit statements including motivation, usefulness, a favorite of method, repeatability, effective feedback, and motivated to study. Answers were based on a three-point Likert scale. Possible selections were “disagree”, “neutral”, and “agree” ranging from 1 which means “disagree” to 3 which means “agree”.

The questionnaire used in our study, has been published previously by Borim Nejad (Supp. 1). The face and content validity of this questionnaire was determined previously, and its reliability was 0.9 through retesting [18].

**Fig. 2:**
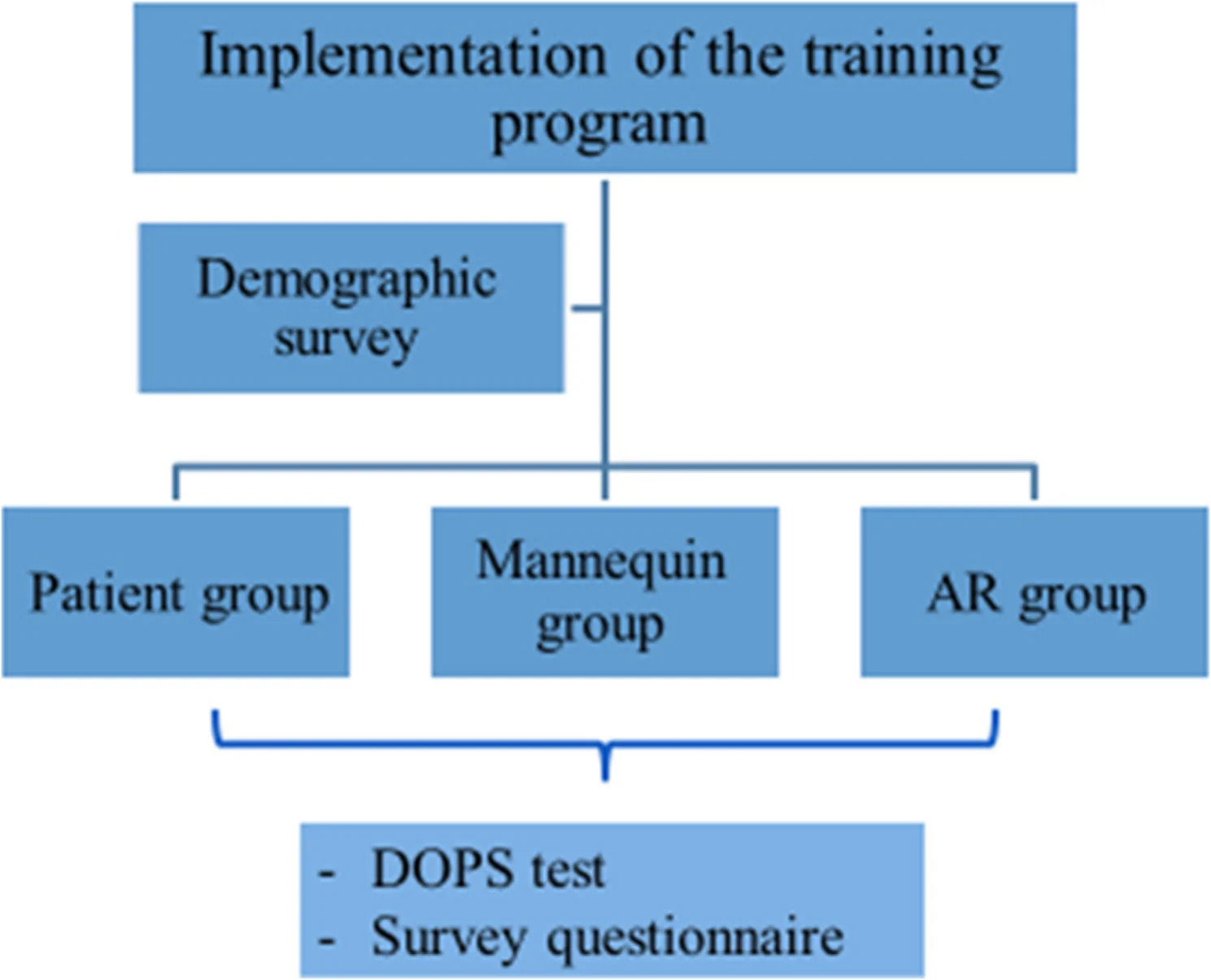
Overview of the design of this study

Figure 2 provides a schematic overview of the design of this study.

### Data collection and analysis

The questionnaire was distributed in a paper-based format to the students after they had used the various learning modalities.

## Results

A total of 90 medical students in the cardiovascular disease course participated in this study. The mean age was 21 (0.7) years. Forty-three (47.7%) of the participants were male, and 47 (52.3%) were female.

### DOPS scoring

The results of the descriptive statistics of the scores obtained by the three groups in the DOPS test are listed in Table 1. Accordingly, the VR group, with an average score of 17.5 out of twenty points, obtained the highest score on this test.

**Table 1:** Comparison of DOPS test scores among the study groups

Group	*n*	Mean (SD)	F^*^	Sig.
Real patient	30	15.2(1.89)	17.2	0.00
Mannequin	30	15.3(1.71)		
VR	30	17.5(1.35)		

*ANOVA

The Shapiro‒Wilk test was used to examine the normality of the distribution, and the results indicated that the distributions were normal.

The results of the ANOVA test revealed a significant difference in the test scores of the three groups. Tukey’s post hoc test revealed a significant difference between the scores of the VR group with real patients and those of the mannequin groups. There was no significant difference between the scores of the real patient group and the mannequin groups (Table 2).

**Table 2:** Tukey post hoc test of the DOPS results

Groups		Mean Difference	Sig.
Real patient	Mannequin	0.06	0.98
	VR	2.23	< 0.005
Mannequin	Real patient	0.06	0.98
	VR	2.16	< 0.005
VR	Real patient	2.23	< 0.005
	Mannequin	2.16	< 0.005

### Satisfaction

The findings revealed that students’ satisfaction in the VR group was greater than that in the other two groups. Given the normality of the distribution, a parametric ANOVA test was used for analytical statistics. These results revealed a significant difference in the test scores among the three groups (Table 3).

**Table 3:** Comparison of satisfaction scores among groups

Group	Mean	St. dev.	F^*^	Sig.
Real patient	1.74	0.24	79.6	0.00
Mannequin	1.79	0.18		
VR	2.35	0.18		

* ANOVA

Tukey’s post hoc test revealed a significant difference between the scores of the VR group with real patients and those of the mannequin groups. There was no significant difference between the scores of the real patient group and the mannequin group (Table 4).

**Table 4:** Tukey post hoc test results

Groups		Mean Difference	Sig.
real patient	Mannequin	0.05	0.58
	VR	0.61	0
Mannequin	Real patient	0.05	0.58
	VR	0.56	0
VR	Real patient	0.61	0
	Mannequin	0.56	0

## Discussion

The present study aimed to compare the effects of electrocardiography lead placement training with three training methods, namely, mannequins, patients, and virtual reality, on the knowledge, performance, and satisfaction of medical students in the Faculty of Medicine of Alborz University of Medical Sciences. The results of the present study revealed a significant difference in DOPS test scores between the VR group with real patients and the mannequin group, whereas there was no significant difference in test scores between the real patient group and the mannequin group. This result indicates that there was a difference in VR group scores due to the use of VR.

This result is consistent with those of numerous studies [19–22]. The higher scores in the virtual reality group may be due to the greater need for a three-dimensional understanding of anatomically contiguous areas. Since anatomy books and atlases cannot teach three-dimensional information, which is especially important in regional anatomy and contiguous areas, virtual reality, with its great capabilities in this field, can be very useful for learners in understanding this concept [23, 24]. The other reason for this could be that the software provides feedback to the learner, allowing for repetition and error correction, which are advantages of the software. Similar studies also indicate greater learner consent with virtual reality-assisted learning [21, 24].

VR technology for anatomy education may also be an excellent resource for teachers where there are cultural or religious beliefs that limit the touch of patients or in institutions that may lack real patients. Learning via virtual reality in an environment that simulates the clinical environment increases student satisfaction by reducing generalized anxiety. In such a comprehensive educational environment, there is no fear of errors or patient reactions, which increases self-confidence and satisfaction [15, 21, 25–27]. In addition, the enjoyable nature and potential for gamification of VR encourage engagement and independent learning. Sometimes the VR method is cheaper than other modalities such as specialized mannequins. Also, the headsets and glasses are usually the most expensive part of VR and the desktop version is cheaper.

VR can provide a clinical scenario in a small space with less than a few minutes of setup. VR scenarios are repeatable. This allows learners to make mistakes safely and then learn through deliberate practice to improve performance.

Additionally, simulators, which use clinical images, videos, and animations and the opportunity to perform repeated exercises and sometimes provide feedback, make students prefer learning with simulators over traditional models [28]. Previous studies have cited its inclusive approach as one of the reasons for greater student satisfaction with virtual reality-assisted teaching [29–33].

Despite these advantages, VR simulations are not suitable for every possible educational opportunity. Additionally, physical simulation should not replace clinical training. VR is just a technology used to deliver a learning technique. Some studies that used VR headsets, have reported adverse effects such as headaches, dizziness, and blurred vision in VR participants [20, 29].

## Limitations of this study

This study had three limitations. First, the sample size was small. Second, the data was collected at a single time point in a single site. Third, this study reflects the views of students from one university in Iran. All of these may affect the validity and accuracy of the relationships between variables such as difficulty. We intend to address this concern in future research by using multiple time points and multiple data sources to further validate our findings.

## Conclusion

The use of simulation technology creates a very good situation for the learner so that the student can observe and act on variables, processes, and procedures in an active situation and interact and react with the environment. This leads to increased experience and can reduce medical errors, which are always major issues in the field of treatment. One way to reduce these errors is to increase the practical training of students, along with improving the quality of education. Given the limited number of cadavers in medical schools and the overcrowding of patients and trainees in teaching hospitals, the need to use simulations and create high-quality virtual spaces is a feasible solution to increase patient health and safety. The limitations of the present study include the small number of students and the short duration of the training.

## Supplementary Information

Supplementary Material 1.

## Data Availability

The datasets supporting the conclusions of this article are available from the corresponding author upon reasonable request.

## Abbreviations

VR: Virtual Reality
DOPS: Direct Observation of Procedural Skills
3D: Three dimensional
