# Supplementary material for: Boosting electrocardiography leads location training through virtual reality simulation: effects on skill performance and learner satisfaction
Source: BMC Med Educ. 2026 Jul 3;26:1516. doi: 10.1186/s12909-026-09854-9 (PMC13602544; doi:10.1186/s12909-026-09854-9)
Supplement: Supplementary file 1 [file 12909_2026_9854_MOESM1_ESM.pdf]

No.	object	Agree 3	Neutral 2	Disagree 1
**1**	I liked this teaching method			
**2**	My information needs were better met			
**3**	The knowledge and skills I gained through this method are useful in my career.			
**4**	This way, I can get a better score			
**5**	Learning with this method happens in a shorter time			
**6**	Learning is better this way			
**7**	This method increases my motivation to learn			
**8**	This method increased my interst			
**9**	This method strengthened my clinical judgment			
**10**	I understood surface anatomy better			
**11**	This method increased my clinical skills			
**12**	This method strngthened my clinical skills			
**13**	This method increased my decision making power			
**14**	With this method I gained a holistic view of the patient			
**15**	Overall, I am satisfied with this method			
**16**	I recommend implementing this method in other courses			
